# High-Temperature Compression Behaviors and Constitutive Models of a 7046-Aluminum Alloy

**DOI:** 10.3390/ma16196412

**Published:** 2023-09-26

**Authors:** Daoguang He, Han Xie, Yongcheng Lin, Zhengbing Xu, Xianhua Tan, Gang Xiao

**Affiliations:** 1 School of Mechanical and Electrical Engineering, Central South University, Changsha 410083, China; 213711034@csu.edu.cn (H.X.); yclin@csu.edu.cn (Y.L.); xg_hnu@163.com (G.X.); 2 State Key Laboratory of Precision Manufacturing for Extreme Service Performance, Changsha 410083, China; 3 Guangxi Key Laboratory of Processing for Non-Ferrous Metals and Featured Materials, Nanning 530004, China; 51happiness123@163.com

**Keywords:** high-temperature compression behaviors, 7046-aluminum alloys, constitutive models, microstructure evolution

## Abstract

High-temperature forming behaviors of a 7046-aluminum alloy were investigated by hot compression experiments. The microstructural evolution features with the changes in deformation parameters were dissected. Results indicated the formation of massive dislocation clusters/cells and subgrains through the intense DRV mechanism at low compression temperature. With an increase in deformation temperature, the annihilation of dislocations and the coarsening of subgrains/DRX grains became prominent, due to the collaborative effects of the DRV and DRX mechanisms. However, the growth of subgrains and DRX grains displayed the weakening trend at high strain rates. Moreover, two constitutive models involving a physically based (PB) model and a gate recurrent unit (GRU) model were proposed for predicting the hot compression features. By validation analysis, the predicted values of true stress perfectly fit with the experimental data, indicating that both the proposed PB model and the GRU model can accurately predict the hot compression behaviors of 7046-aluminum alloys.

## 1. Introduction

Aluminum alloys have attracted considerable attention for military aircraft, automobiles and weapons because of their preeminent mechanical properties and corrosion resistance [1,2,3,4,5,6]. Due to the addition of numerous alloying elements, the aluminum alloys are frequently subjected to various microstructural evolution mechanisms [7,8], which results in the appearance of complicated hot flow features [9,10,11,12]. Hence, further research into the flow characteristics and microstructural evolution features of aluminum alloys in hot deforming is essential.

Researchers have systematically investigated how these microstructure evolution characteristics were conducive to regulating the hot flow features of aluminum alloys [13,14,15]. First, the evolution of dislocations and the coarsening behaviors of subgrains were deeply revealed in numerous studies [16,17,18]. Moreover, the development features in dynamic recrystallization (DRX) grains and the extension of grain boundaries in aluminum alloys were researched [19,20,21,22]. In addition, the precipitation behaviors and the dissolution features of phase were dissected [23,24].

Recently, many constitutive models were established or improved to capture the hot tensile/compression flow features of alloys [25,26,27,28,29,30,31]. For instance, the evolution features of flow behaviors with the Zener–Hollomon (Z) parameters were explored, and various phenomenological equations were constructed for depicting hot deforming features in aluminum alloys [32,33,34]. Meanwhile, numerous physically based (PB) equations were constructed to predict the hot flow stress and microstructures for aluminum alloys [35,36,37,38], Ni-based alloys [39] and ultrahigh-strength steels [40,41]. In addition, various artificial neural network (ANN) models [42,43,44] containing BP models [45,46,47] and long short-term memory (LSTM) models [48,49] have been used to predict hot flow behaviors in aluminum alloys. Another preferred LSTM model, called a gate recurrent unit (GRU) neural network, was developed from ANN models. The GRU model contains fewer parameters than the LSTM model, thus boosting its computing efficiency while simultaneously improving the prediction accuracy [50,51]. Nowadays, as GRU theory continues to advance, GRU models are extensively used in various domains, including data modeling [52], text classification [53] and electric power load forecasting [54].

Although numerous investigations have focused on exploring hot deforming behaviors in aluminum alloys, the complicated microstructure evolution mechanisms and hot flow behaviors need to be further explored. In this investigation, the evolution characteristics of true stress and microstructures for the 7046-aluminum alloy are explored. Especially, the evolution features of dislocation clusters/subgrains and DRX nucleating mechanisms are revealed. Additionally, both a PB model and a GRU model were set up for describing the hot compression stress–strain characteristics in the 7046-aluminum alloy. Simultaneously, the reconstitution capacity of each constitutive model was verified.

## 2. Materials and Experimental Approach

**Figure 1:**
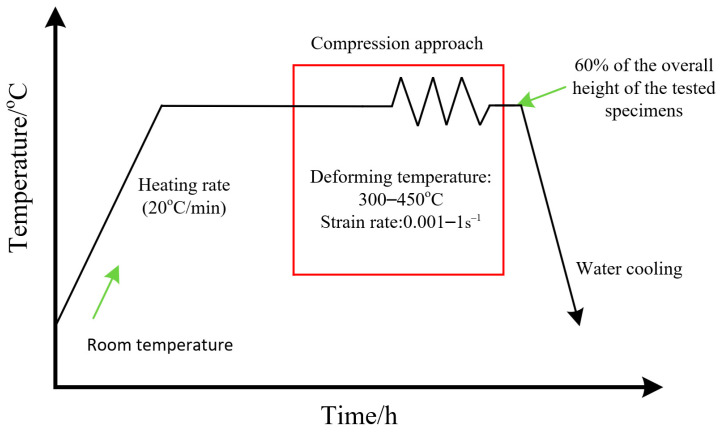
The hot forming steps for a 7046-aluminum alloy.

In the present investigation, a commercial 7046-aluminum alloy with the chemical components (wt.%) of 6.6Zn-1.7Mg-0.25Cu-0.15Zr-Al (Bal) was used. Normal cylindrical specimens (ϕ8 mm × 12 mm) were fabricated from the 7046-aluminum alloy plate. Hot compression experiments were executed on a Gleeble-3500 device (Poestenkill, NY, USA). The details of the hot compression process are revealed in Figure 1. The specimens were heated to setting forming temperature (T) and then held 360 s. The hot compression of each sample was implemented at a constant value of T and strain rate ($\dot{\varepsilon }$). The values of T were selected to be from 300 °C to 450 °C with intervals of 50 °C. Moreover, the values of $\dot{\varepsilon }$ were set as 0.001–1 s^−1^, and the final strain was 0.92. When the final strain was reached, the test specimens were quickly cooled by water. 

Backscattering electron microscopy (EBSD) (Velocity Super, EDAX-Ametek, Pleasanton, CA, USA) was utilized to examine the initial microstructure of the investigated 7046-aluminum alloy. In order to analyze the microstructural evolution features in hot compression, optical microscopy (OM) (Olympus DSX500, Tokyo, Japan) and a transmission electron microscope (TEM) (Tecnai G2 F20, FEI Company, USA) were used. Specimens for OM observations were first mechanically ground and polished, followed by etching with a Keller reagent [4]. For the TEM and EBSD observations, the pieces were mechanized along the axial direction of hot compressed samples and substantially polished in a solution (15 mL ${\mathrm{HClO}}_{4}$ and 135 mL ${\mathrm{CH}}_{3}{\mathrm{CH}}_{2}\mathrm{OH}$).

**Figure 2:**
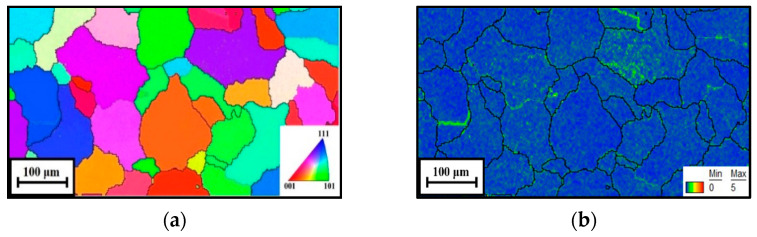
The EBSD analysis of the initial microstructure: (**a**) IPF image; (**b**) KAM image.

Figure 2 displays the EBSD images of the initial microstructure. Obviously, some equiaxed grains can be detected (Figure 2a). Moreover, almost all the grains are covered with the color blue, which demonstrates that few substructures remained (Figure 2b). 

## 3. High-Temperature Compression Characteristics

### 3.1. Analysis of Hot Compression Flow Curves

**Figure 3:**
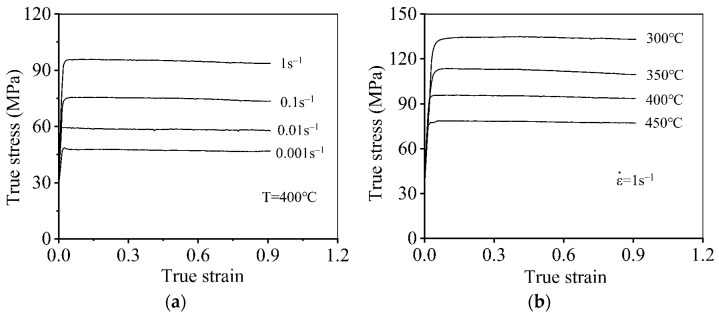
Typical hot-compressed features of the 7046-aluminum alloy at: (**a**) T=400 °C; (**b**) $\dot{\varepsilon }=1$ s^−1^.

Figure 3 reveals the flow behaviors of the 7046-aluminum alloy in hot compression. The similar evolution tendency in all the curves revealed that true stresses swiftly increase because of the acute work hardening (WH) effect correlated with the evolution of substructures [18]. When the hot compression continues, a decrease in true stress occurs due to the cooperation effects of the dynamic recovery (DRV) and dynamic recrystallization (DRX) [22]. Furthermore, it was revealed that true stresses are reduced with the decreasing strain rate ($\dot{\varepsilon }$), as seen in Figure 3a. This is because an extended deformation time is provided to the development of subgrains and DRX grains with the decrease in $\dot{\varepsilon }$. Thus, the softening effects associated with the mechanisms of DRV and DRX are enhanced, resulting in the decrease of true stresses. Moreover, the true stresses display the decreasing trend with the increase of deformation temperature (T), as seen in Figure 3b. One cause is that the DRV mechanism connected with the annihilation of dislocation clusters and the development of subgrains is improved at higher T. Moreover, the progression of DRX features also displays the reinforcing effect with the elevated T. Hence, the decrease of the true stress emerges.

### 3.2. Microstructure Evolution Mechanisms

**Figure 4:**
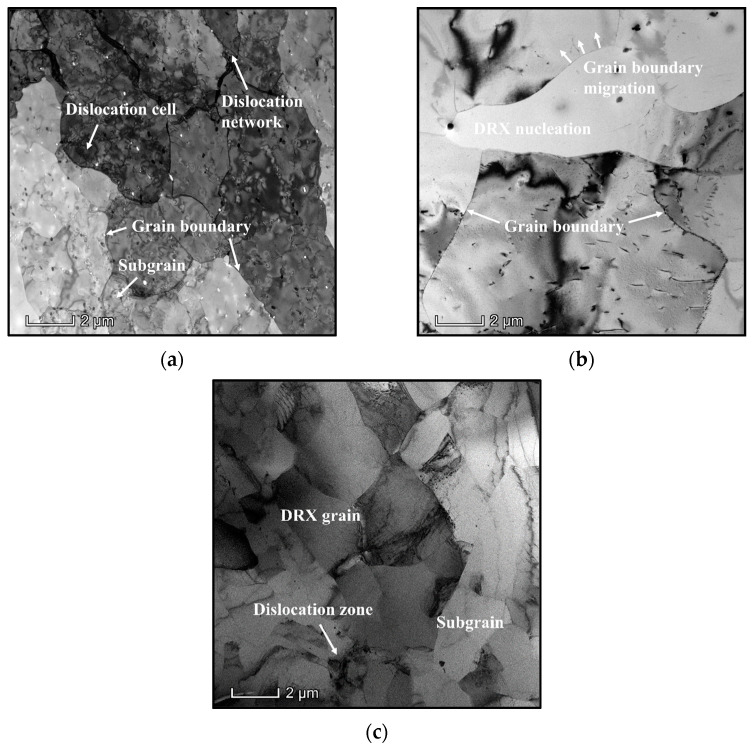
TEM maps at: (**a**) 300 °C/0.001 s^−1^; (**b**) 400 °C/0.001 s^−1^; (**c**) 400 °C/1 s^−1^.

The typical evolution features of dislocations and subgrains with the variation of compressed parameters were explored by TEM observation (Figure 4). Clearly, some dislocations are generated and accumulated to form the dislocation networks/cells at 300 °C/0.001 s^−1^, as seen in Figure 4a. Concurrently, many subgrains can also be observed. With the increase of T, the DRX grains become apparently coarsening and the annihilation of substructures are enhanced (Figure 4b). It is because that the diffusion of alloy atoms/vacancies are sensibly reinforced at higher T [15], which enhances the consumption of substructures through the intense DRV mechanism. Furthermore, the expansion rate of grain boundaries is becoming strengthened with the increase of T [19], and then the coarsening capacity of DRX grains is significantly enhanced. With the $\dot{\varepsilon }$ increasing to 1 s^−1^, the nucleation of high-density substructures containing dislocation clusters/arrays and subgrains can be observed (Figure 4c). Simultaneously, almost all DRX grains at 400 °C/0.001 s^−1^ become coarsening, compared to that seen at 400 °C/1 s^−1^. The predominant cause is that the shorter deforming time for the dislocations’ climbing/interaction and the propagation of grain boundaries can be ensured at higher $\dot{\varepsilon }$ [29,39]. 

**Figure 5:**
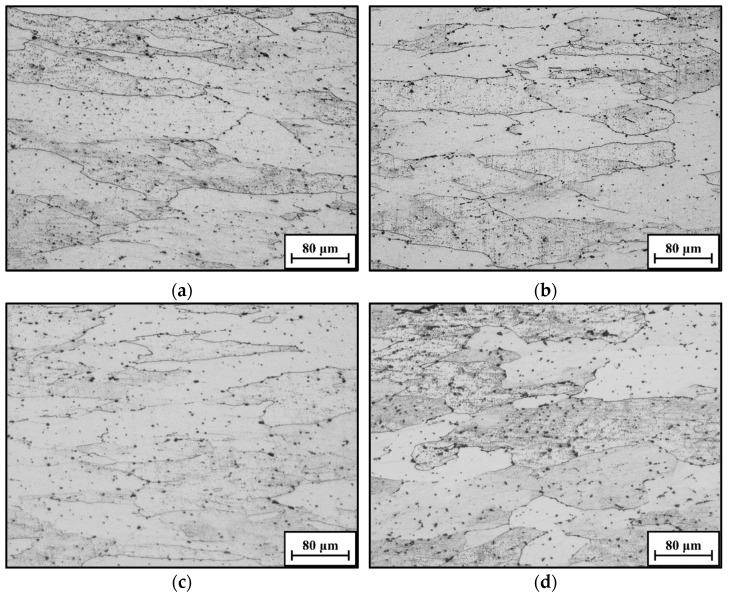
OM maps at: (**a**) 400 °C/1 s^−1^; (**b**) 450 °C/1 s^−1^; (**c**) 450 °C/0.01 s^−1^; (**d**) 450 °C/0.001 s^−1^.

Figure 5 reveals the typical evolution features of grain morphology under different forming conditions. Clearly, the almost original grains reveal elongated characteristics at 400 °C/1 s^−1^, and several fine DRX grains gather near them (Figure 5a). With the T increasing to 450 °C, the nucleation and coarsening behaviors of DRX grains become apparent (Figure 5b). The main cause is that the significant development of substructures appears at high T, which promotes the formation of DRX nucleus. Meanwhile, the pinning effect for the grain boundaries becomes weakened due to the annihilation of high-density substructures, which is beneficial for DRX grain coarsening. Moreover, the DRX grains also display the coarsening tendency with the decrease of $\dot{\varepsilon }$ (Figure 5c,d). That is because the deforming time for the consumption of substructures through the annihilation of dislocations and the diffusion of vacancies is extended, which reduces the resistance to the grain boundary migration. Thus, the DRX grains reflect the outstanding growth behaviors at lower $\dot{\varepsilon }$. 

## 4. Constitutive Models for Predicting High-Temperature Compression Behavior

### 4.1. A Proposed PB Model

#### 4.1.1. Modeling the Flow Stress Induced by Work Hardening and Dynamic Recovery

Due to the interaction of WH and DRV mechanisms, the flow stress in the initial period of hot compression displays a sharply increasing tendency (Figure 3), and its evolution behavior can be described utilizing the Estrin–Mecking model [55],

(1) $$\sigma_{\mathrm{rec}}={\left[\sigma_{\mathrm{sat}}^{2}+\left(\sigma_{y}^{2}-\sigma_{\mathrm{sat}}^{2}\right)\exp(-2\psi \varepsilon )\right]}^{1/2}$$

where $\sigma_{\mathrm{rec}}$ is flow stresses, $\sigma_{y}$ is the yield stress, $\sigma_{\mathrm{sat}}$ is the saturation stress, ε is strain, and ψ is the material constant.

Commonly, the evolution characteristics of $\sigma_{y}$ and $\sigma_{\mathrm{sat}}$ for alloys in hot deforming are tightly associated with the Zener–Hollomon (*Z*) parameter [55]. Accordingly, the *Z* parameter is usually ascertained as [32],

(2) $$Z=\dot{\varepsilon }\exp\left(\frac{Q}{RT}\right)$$

(3) $$\dot{\varepsilon }=\left\{\begin{array}{l} A{\left[\sinh(\alpha \sigma )\right]}^{n}\exp\left(-\frac{Q}{RT}\right),\mathrm{for}\;\mathrm{all}\;\sigma \\ B\sigma^{n^{\prime }}\exp\left(-\frac{Q}{RT}\right),\alpha \sigma <0.8\\ C\exp(\beta \sigma )\exp\left(-\frac{Q}{RT}\right),\alpha \sigma >1.2 \end{array}\right.$$

where the Q is the deforming activated energy, R is the gas constant (8.314 J/mol/K), and A, B, C, n, $n^{\prime }$ and β are material constants.

The peak stress ($\sigma_{p}$) is commonly used to evaluate the material constants in Equation (3) [56]. The $\sigma_{p}$ is substituted into Equation (3) and it is given as [57],

(4) $$\left\{\begin{array}{l} \dot{\varepsilon }=B^{\prime }\sigma_{p}^{n^{\prime }},\alpha \sigma_{p}<0.8\\ \dot{\varepsilon }=C^{\prime }\exp(\beta \sigma_{p}),\alpha \sigma_{p}>1.2 \end{array}\right.$$

where $B^{\prime }$ and $C^{\prime }$ are material constants. 

**Figure 6:**
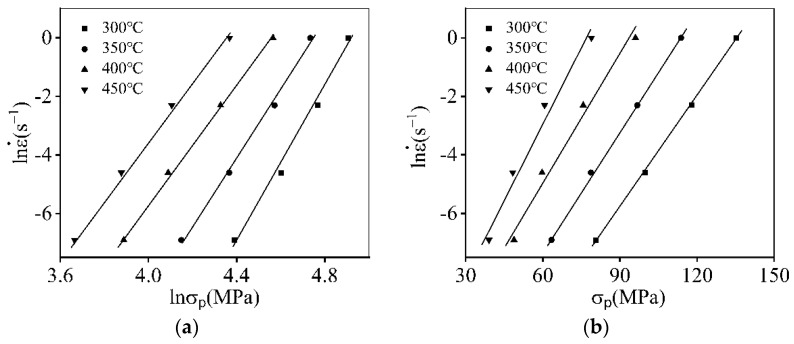
The plots of: (**a**) $\ln\dot{\varepsilon }-\ln\sigma_{p}$; (**b**) $\ln\dot{\varepsilon }-\sigma_{p}$.

According to Equation (4), the values of $n^{\prime }$ and β are identified by the correlations of $\ln\dot{\varepsilon }-\ln\sigma_{p}$ and $\ln\dot{\varepsilon }-\sigma_{p}$, respectively. For the researched 7046-aluminum alloy, the variation characteristics of $\ln\dot{\varepsilon }-\ln\sigma_{p}$ and $\ln\dot{\varepsilon }-\sigma_{p}$ are revealed in Figure 6. Then, the mean values of $n^{\prime }$ and β are identified as 11.239 and 0.146, respectively. Accordingly, the $\alpha =\beta /n^{\prime }$ = 0.0130 ${\mathrm{MPa}}^{-1}$.

Moreover, the Q in Equation (3) is identified by [58],

(5) $$Q=R{\left\{\frac{\partial \ln\dot{\varepsilon }}{\partial \ln\left[\sinh\left(\alpha \sigma_{p}\right)\right]}\right\}}_{T}\cdot {\left\{\frac{\partial \ln\left[\sinh\left(\alpha \sigma_{p}\right)\right]}{\partial (1/T)}\right\}}_{\dot{\varepsilon }}$$

**Figure 7:**
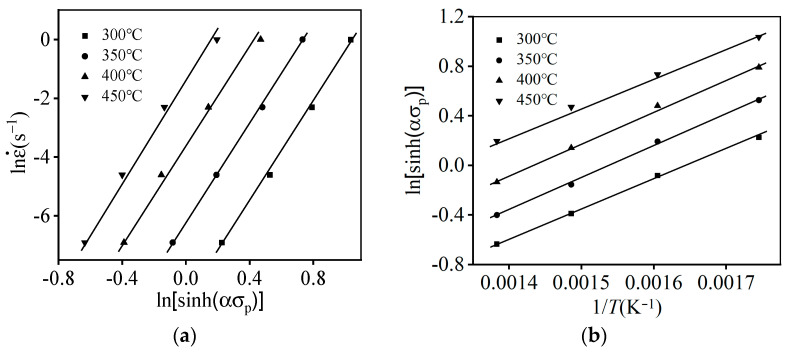
Correlations of: (**a**) $\ln\dot{\varepsilon }-\ln[\sinh(\alpha \sigma_{p})]$; (**b**) $\ln[\sinh(\alpha \sigma_{p})]-1/T$.

Figure 7 reveals the variation characteristics of $\ln\dot{\varepsilon }-\ln[\sinh(\alpha \sigma_{p})]$ and $\ln[\sinh(\alpha \sigma_{p})]-1/T$. Through the linear-fitting computation, the *Q* is estimated as 170.260 kJ/mol, which is similar to that of other Al-Zn-Mg-Cu alloys [27].

**Figure 8:**
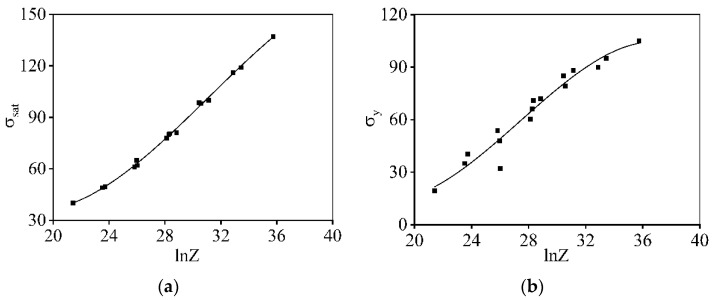
Relation of: (**a**) $\sigma_{\mathrm{sat}}-\ln Z$, (**b**) $\sigma_{y}-\ln Z$.

In addition, the values of $\sigma_{\mathrm{sat}}$ for alloys in hot deforming are usually determined by the θ=dσ/dε and σ curve [59]. Figure 8 reveals the variations in $\sigma_{\mathrm{sat}}$ and $\sigma_{y}$ with the *Z* parameters. As revealed in Figure 8a, the correlation of $\sigma_{\mathrm{sat}}$ and lnZ exhibits nonlinear characteristics. Concurrently, the evolution characteristics of $\sigma_{y}$ over the *Z* parameter are revealed in Figure 8b. Based on the polynomial fitting method, the variations in $\sigma_{\mathrm{sat}}$ and $\sigma_{y}$ with ln*Z* are identified, respectively, as listed in Equations (6) and (7).

(6) $$\sigma_{\mathrm{sat}}=369.837-41.726\ln Z+1.595{\left(\ln Z\right)}^{2}-0.017{\left(\ln Z\right)}^{3}$$

(7) $$\sigma_{y}=422.452-59.247\ln Z+2.244{\left(\ln Z\right)}^{2}-0.027{\left(\ln Z\right)}^{3}$$

Furthermore, the material constant of ψ in Equation (1) can be determined by

(8) $$\psi =\frac{1}{2\varepsilon }\ln\left(\frac{\sigma_{\mathrm{sat}}^{2}-\sigma_{y}^{2}}{\sigma_{\mathrm{sat}}^{2}-\sigma_{\mathrm{rec}}^{2}}\right)$$

**Figure 9:**
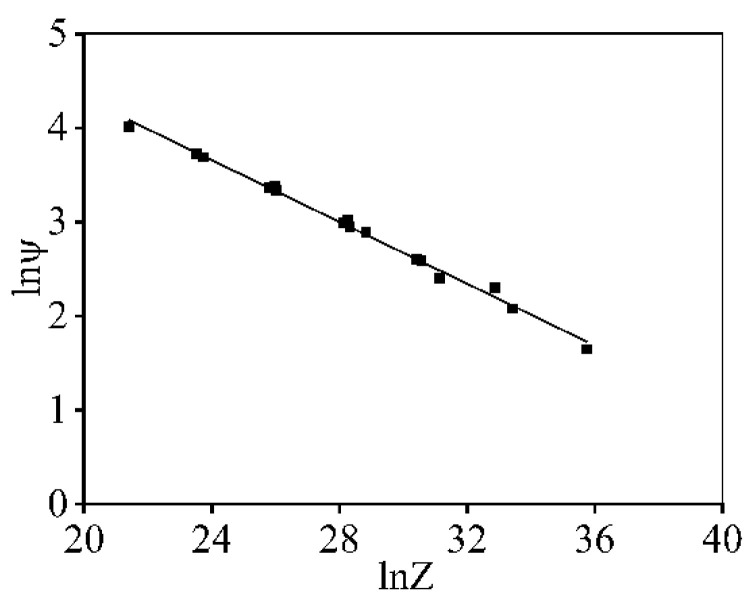
Relation between ψ and ln*Z*.

Using the experimental data, the relations of lnψ and ln*Z* can be determined, as revealed in Figure 9. Clearly, the variation feature of lnψ and ln*Z* displays the linear correlation. Hence, the ψ is identified as

(9) $$\psi =1998.2Z^{-0.164}$$

#### 4.1.2. Modeling the Flow Stress Induced by Work Hardening and DRX

As the DRX is excited, the flow stress descends notably due to the mutual influences of DRX and DRV [22]. Normally, the DRX fraction (X) is identified as [60],

(10) $$X=\frac{\sigma_{\mathrm{rec}}-\sigma_{\mathrm{drx}}}{\sigma_{\mathrm{sat}}-\sigma_{\mathrm{ss}}}$$

where $\sigma_{\mathrm{ss}}$ is steady-state stress. 

Moreover, the X is intimately influenced by the critical strain ($\varepsilon_{c}$) and can be identified as [60],

(11) $$X=1-\exp\left[-c_{1}{\left(\varepsilon -\varepsilon_{c}\right)}^{c_{2}}\right]$$

where $c_{1}$ and $c_{2}$ are material constants. 

Using Equations (1) and (11), the $\sigma_{\mathrm{drx}}$ is determined as [55],

(12) $$\sigma_{\mathrm{drx}}=\sigma_{\mathrm{rec}}-\left\{1-\exp\left[-c_{1}{\left(\varepsilon -\varepsilon_{c}\right)}^{c_{2}}\right]\right\}\left(\sigma_{\mathrm{rec}}-\sigma_{\mathrm{ss}}\right),\varepsilon \geq \varepsilon_{c}$$

**Figure 10:**
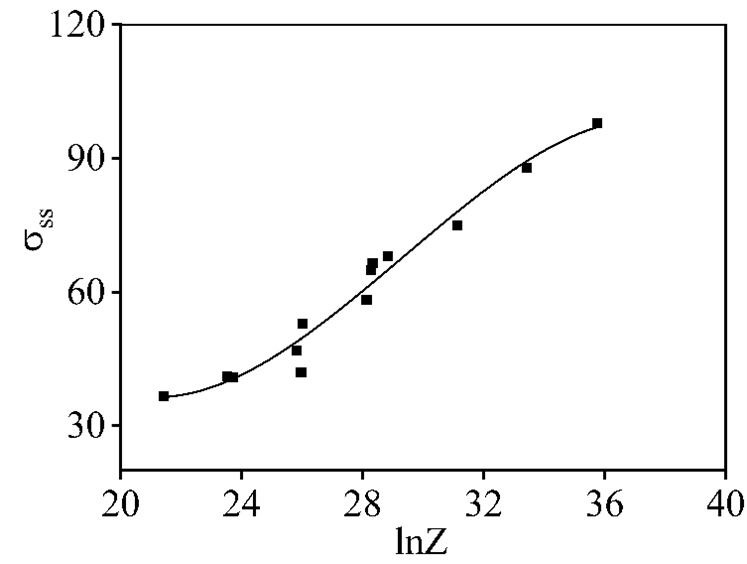
Relation between $\sigma_{\mathrm{ss}}$ and ln*Z*.

Clearly, the values of $\sigma_{\mathrm{drx}}$ are primarily associated with the four factors ($c_{1}$, $c_{2}$, $\varepsilon_{c}$, $\sigma_{\mathrm{ss}}$). Figure 10 reveals the variations in $\sigma_{\mathrm{ss}}$ with the Z parameters. Using the polynomial fitting analysis, the $\sigma_{\mathrm{ss}}$ can be ascertained as

(13) $$\sigma_{\mathrm{ss}}=626.56-68.729\ln Z+2.542{\left(\ln Z\right)}^{2}-0.029{\left(\ln Z\right)}^{3}$$

In addition, for alloys in hot deforming, the correlation between $\varepsilon_{c}$ and peak strain ($\varepsilon_{p}$) is commonly identified as [61],

(14) $$\varepsilon_{c}=S_{c}\varepsilon_{p}$$

where *S*_c_ is the material constant. For Al-Zn-Mg-Cu alloys in hot deforming, the scopes of *S*_c_ are often chosen as 0.6~0.8 [61]. Here, the value of *S*_c_ is selected as 0.8. 

**Figure 11:**
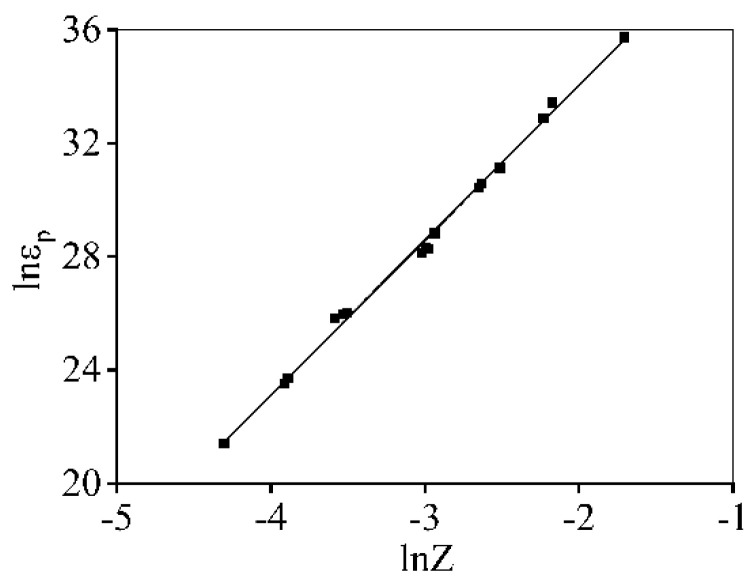
Relation between $\ln\varepsilon_{p}$ and ln*Z*.

Figure 11 reveals the changes of $\varepsilon_{p}$ with *Z* parameters. Using the linear-fitting calculation, the $\varepsilon_{p}$ is determined as

(15) $$\varepsilon_{p}=44.964Z^{5.462}$$

**Figure 12:**
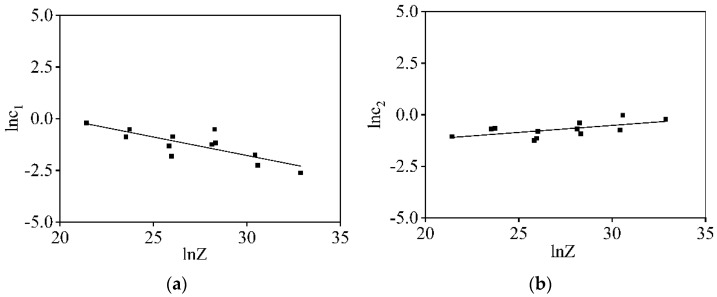
Relations of: (**a**) $\ln c_{1}-\ln Z$; (**b**) $\ln c_{2}-\ln Z$.

Based on Equation (11), the values of two material constants ($c_{1}$ and $c_{2}$) can be identified utilizing linear regression analysis of the $\ln\left(-\ln(1-X)\right)$ − $\ln(\varepsilon -\varepsilon_{c})$ plots. The changes of the material constants ($c_{1}$ and $c_{2}$) with the *Z* parameters are indicated in Figure 12a,b. Then, $c_{1}$ and $c_{2}$ can be determined as

(16) $$c_{1}=36.162Z^{-0.179}$$

(17) $$c_{2}=0.078Z^{0.068}$$

According to the above analysis, the constituted PB model can be summarized as

(18) $$\left\{\begin{array}{l} \sigma_{\mathrm{rec}}={\left[\sigma_{\mathrm{sat}}^{2}+\left(\sigma_{y}^{2}-\sigma_{\mathrm{sat}}^{2}\right)\exp(-2\psi \varepsilon )\right]}^{1/2}\\ \sigma =\sigma_{\mathrm{rec}}-\left\{1-\exp\left[-c_{1}{\left(\varepsilon -\varepsilon_{c}\right)}^{c_{2}}\right]\right\}\left(\sigma_{\mathrm{rec}}-\sigma_{\mathrm{ss}}\right),\varepsilon \geq \varepsilon_{c}\\ \sigma_{\mathrm{sat}}=369.837-41.726\ln Z+1.595{\left(\ln Z\right)}^{2}-0.017{\left(\ln Z\right)}^{3}\\ \sigma_{y}=422.452-59.247\ln Z+2.244{\left(\ln Z\right)}^{2}-0.027{\left(\ln Z\right)}^{3}\\ \sigma_{\mathrm{ss}}=626.56-68.729\ln Z+2.542{\left(\ln Z\right)}^{2}-0.029{\left(\ln Z\right)}^{3}\\ \psi =1998.2Z^{-0.164}\\ c_{1}=36.162Z^{-0.179}\\ c_{2}=0.078Z^{0.068}\\ Z=\dot{\varepsilon }\exp\left(\frac{170260}{RT}\right)\\ \varepsilon_{p}=44.964Z^{5.462}\\ \varepsilon_{c}=0.8\varepsilon_{p} \end{array}\right.$$

In addition, the values of the material constants in Equation (18) are listed in Table 1.

### 4.2. The GRU Machine Learning Model

As analyzed in Section 3.1, the changes in true stress with forming parameters ($T,\dot{\varepsilon }$ and ε) indicate the typical nonlinear characteristics. Due to its superior data-driven performance for modeling multi-factor coupled data, a GRU model was proposed to capture the hot compression features of the researched aluminum alloy. 

**Figure 13:**
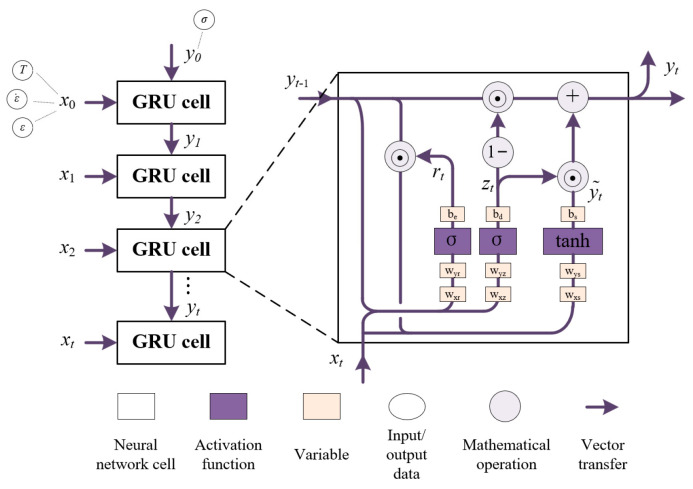
Schematic diagram of GRU cells.

Figure 13 depicts the typical architecture of the GRU model. Commonly, the GRU model can dynamically update the implied state of the following cell in real time based on the historical data of the preceding GRU cell. Generally, the cyclic essence of the GRU model is reflected in the fact that model inputs ($x_{t}$) and the previous output hidden state ($y_{t-1}$) are transferred to the next GRU cell. Hot compression parameters containing $T,\dot{\varepsilon }$ and ε are introduced to the network structure as the inputs, and the calculated results of the GRU cells are σ. 

Normally, GRU cells internally incorporate sophisticated and flexible structures, called gate connections, as shown in Figure 13. The gate connections, predominantly both reset gates ($r_{t}$) and update gates ($z_{t}$), are responsible for the learning and computational processes of the input data in the GRU model. 

Primarily, the reset gates ($r_{t}$) regulate how much state information from the previous state will be ignored in the hidden layer vector [52]. 

Normally, the equation of $r_{t}$ can be expressed as [52],

(19) $$r_{t}=\sigma \left(W_{xr}x_{t}+W_{yr}y_{t-1}+b_{e}\right)$$

where $W_{xr}$ and $W_{yr}$ are neuron connection weights of the reset gate, $b_{e}$ is the bias of the reset gate, and σ(t) is the sigmoid activation function, which can be indicated as

(20) $$\sigma (t)=\frac{1}{1+e^{-t}}$$

where *t* indicates input elements.

Next, the update gate ($z_{t}$) modulates how much state information from the previous implicit layer is maintained to the candidate hidden state. The update gate ($z_{t}$) can be expressed as [51],

(21) $$z_{t}=\sigma \left(W_{xz}x_{t}+W_{yz}y_{t-1}+b_{d}\right)$$

where $W_{xz}$ and $W_{yz}$ are neuron connection weights of the update gate, and $b_{d}$ is the bias of the update gate.

The candidate hidden state (${\tilde{y}}_{t}$) contains the most recent time step information, and can be updated as follows [50]:

(22) $${\tilde{y}}_{t}=\tanh\left(W_{xs}\cdot x_{t}+W_{ys}\cdot (r_{t}⊙y_{t-1})+b_{s}\right)$$

where the symbol ⊙ is multiplication of elements. tanh(t) is the hyperbolic tangent function, which can be represented as

(23) $$\tanh(t)=\frac{e^{t}-e^{-t}}{e^{t}+e^{-t}}$$

In conclusion, the final update gets the hidden state of the current time step and can be expressed as [53],

(24) $$y_{t}=\left(1-z_{t}\right)⊙y_{t-1}+z_{t}⊙{\tilde{y}}_{t}$$

Prior to training the GRU model, the input data is normalized and the normalization expression can be revealed as

(25) $${X^{\prime }}_{in}=\frac{X_{in}-X_{in}^{\left(\min\right)}}{X_{in}^{\left(\max\right)}-X_{in}^{\left(\min\right)}}$$

where ${X^{\prime }}_{in}$ is the normalized data, $X_{in}$ is the original data, and $X_{in}^{\left(\max\right)}$ and $X_{in}^{\left(\min\right)}$ are the maximum and minimum values of $X_{in}$, respectively. After the normalization of input data, the corresponding values ($T^{\prime },{\dot{\varepsilon }}^{\prime },\varepsilon^{\prime }$) are collapsed into sequences as training inputs. Here, the experimental data utilization was split at random into 80% for model training and the remaining 20% for model testing and validation.

Commonly, the excellent predictive accuracy and rapid response of the GRU model are impacted by the diverse hyperparameters. The numbers of hidden layers and neurons in the hidden layers, the initial learning rate and the batch size are the main alterable hyperparameters of the GRU model. Various hidden layers can promote model accuracy, but it has been demonstrated that too many hidden layers will lead to overfitting [51]. In this paper, the number of hidden layers was set to three, balancing accuracy and overfitting. Additionally, the number of neurons in the hidden layers was set to decrease layer by layer. The learning rate and the batch size of the GRU model play the critical roles [62]. A higher initial learning rate or batch size allows for quicker training but may lead to a less accurate and unstable training. For further probing the connection between the three hyperparameters and the forecast results, an orthogonal experiment table with three factors and four levels was devised, as shown in Table 2. Based on that orthogonal experiment table, the calculated results of $L_{16}(3^{4})$ are shown in Table 3. Here, the model accuracy is assessed by the validation loss, which is characterized using the mean squared error (MSE). The MSE equation is,

(26) $$\mathrm{MSE}=\frac{1}{N}\sum_{i=1}^{N}{\left(E_{i}-P_{i}\right)}^{2}$$

where N is the number of values, $E_{i}$ is the experimental stress, and $P_{i}$ is the predicted stress.

**Figure 14:**
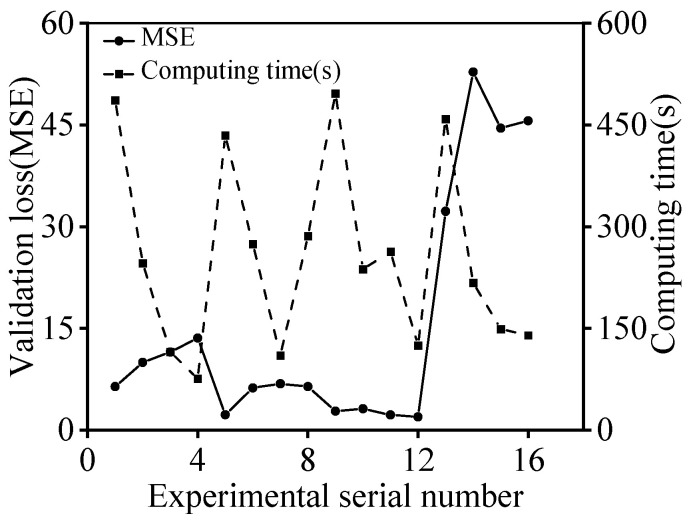
Variations in MSE and computing time under different hyperparameters.

Figure 14 describes the variations of MSE and computing time under different hyperparameters. On the one hand, the MSE of the GRU model decreases to a minimal value when the initial learning rate increases from 0.001 to 0.01. On the other hand, the MSE value begins to violently fluctuate as the initial learning rate ascends or declines; thus, the perfect learning rate can be set at 0.01. Meanwhile, as the batch size increases, the computation time can be effectively reduced without affecting the accuracy of the GRU model. Hence, the batch size was determined as 64. Summarily, the values of the initial learn rate, batch size and numbers of neurons in hidden layers were selected as 0.01, 64 and 100/80/60, respectively.

### 4.3. Comparison and Validation

**Figure 15:**
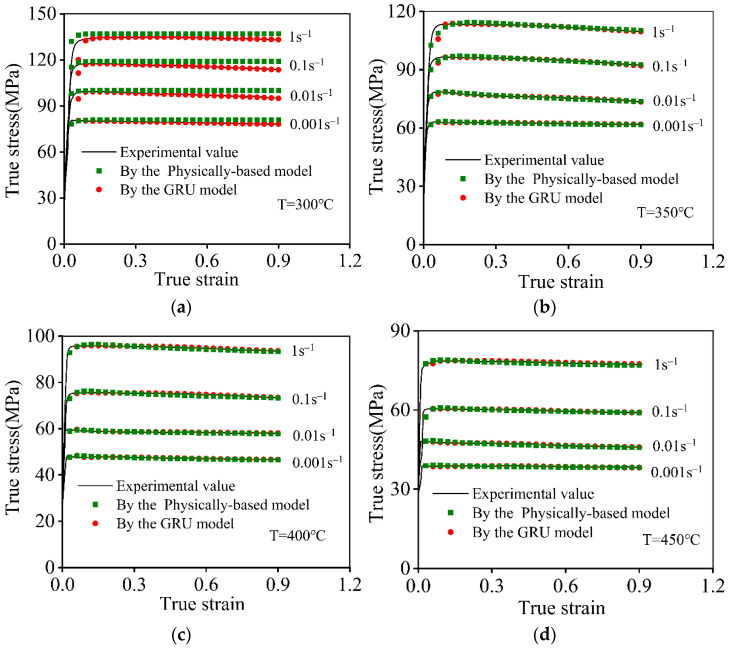
Comparative analysis of the tested stresses and the forecasted results at: (**a**) 300 °C; (**b**) 350 °C; (**c**) 400 °C; (**d**) 450 °C.

Based on the proposed PB and GRU models, the hot compression stress–strain features for the 7046-aluminum alloy are reconstituted. Figure 15 depicts the comparison analysis of the flow stress predicting ability of the proposed PB and GRU models. Clearly, the tested hot compression stresses match very well with the values predicted by the GRU model. The primary reason was that the GRU model has an outstanding ability to describe nonlinear correlations between input values and output results [54]. Moreover, a disparity between the tested compression stresses and the predicted ones utilized by the PB model was discovered at 300 °C (Figure 15a). However, the compression stresses preeminently fit the forecasting results as the T reached 350 °C or above (Figure 15c,d). The primary reason for these phenomena was that the microstructural evolution mechanisms are intimately connected with the compression parameters [23]. The DRV behavior acts as the predominant softening mechanism in the hot deforming process of a 7046-aluminum alloy when the T is less than 350 °C. Nevertheless, the DRX characteristics are activated when the T reaches 350 °C or above, and they evolve into the predominant softening mechanism for the 7046-aluminum alloy. Because of the appearance of varying microstructural evolution mechanisms, the variations in compression stresses over deforming parameters exhibit complicated and non-linear features. Thus, the forecasting accuracy of the PB model is relatively lower than that of the GRU model. 

In addition, the average absolute relative error (AARE) and the correlation coefficient (C_C_) were calculated to further estimate the forecasting ability of the PB model and the GRU model. The values of AARE and C_C_ are determined as

(27) $$\mathrm{AARE}(\%)=\frac{1}{N}\sum_{i=1}^{N}\left|\frac{E_{i}-P_{i}}{E_{i}}\right|\times 100\%$$

(28) $$C_{C}=\frac{\sum_{i=1}^{N}\left(E_{i}-\bar{E}\right)\left(P_{i}-\bar{P}\right)}{\sqrt{\sum_{i=1}^{N}{\left(E_{i}-\bar{E}\right)}^{2}\sum_{i=1}^{N}{\left(P_{i}-\bar{P}\right)}^{2}}}$$

where N is the number of the data; $E_{i}$ and $P_{i}$ are the testing stresses and forecasting ones, respectively; and $\bar{E}$ and $\bar{P}$ are the mean values of $E_{i}$ and $P_{i}$, respectively. 

**Figure 16:**
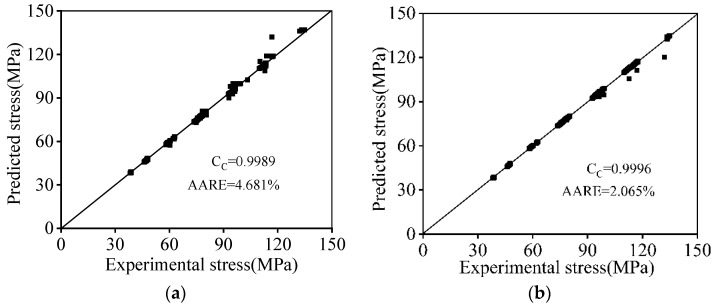
Relationships of tested compression stresses and values forecasted by (**a**) the PB model; (**b**) the GRU model.

The further correlation analyses of the tested stresses and the results forecast by the constructed PB and GRU models are shown in Figure 16. The values of the AARE for the PB model and the GRU model are determined to be 4.681% and 2.065%, respectively, which are less than 5.0%. Concurrently, the C_C_ values for the PB model and the GRU model are determined as 0.9989 and 0.9996, respectively. The relatively small AARE and large C_C_ show that both the PB model and the GRU model enjoy sufficient precision to capture the hot compression characteristics. Additionally, it can also be inferred that the proposed GRU model possesses better forecasting ability for the hot compression behaviors of a 7046-aluminum alloy than that of the PB model. 

Similar research on the hot tensile characteristics of the 7046-aluminum alloy can be found in the authors’ previous study [48]. An improved Hensel–Spittel–Carofalo (HSC) model and a long short-term memory (LSTM) model are established to reconstruct the high-temperature tensile features. The C_C_ and AARE values of all four models are listed in Table 4. Comparing the four constitutive models, it is clear that the ANN models, including the LSTM model and the GRU model, had better forecast performance than the PB model and the HSC model. However, the PB model has the superior capability for predicting the hot forming behaviors for the 7046-aluminum alloy.

## 5. Conclusions

Hot compression characteristics and microstructural evolution mechanisms for the 7046-aluminum alloy were revealed. Some prominent conclusions of this study are as follows:

(1) Hot flow characteristics of a 7046-aluminum alloy are intimately associated with compression parameters. As compression temperature ascends or the strain rate descends, the flow stresses display the dominant reducing characteristic.
(2) Microstructural evolution characteristics are intensely influenced by the compression parameters. The formation/interaction of substructures exhibits the intensified trend, while the extension of DRX grain boundaries becomes inhibited at a high strain rate or low compression temperature.
(3) A PB model and a GRU model were proposed to describe the hot compression behaviors of the 7046-aluminum alloy. The relatively smaller AARE and larger C_C_ demonstrated that both the proposed PB model and the GRU model can precisely achieve the reconstitution of hot compression behaviors in the 7046-aluminum alloy.

## Figures and Tables

**Table 1:** The material constants of the 7046-aluminum alloy.

Number	Notation	Values	Note
1	Q	170.260 kJ/mol	deforming activated energy
2	R	8.314 J/mol/K	gas constant
3	$n^{\prime }$	11.239	material constant
4	β	0.146	material constant
5	α	0.0130 ${\mathrm{MPa}}^{-1}$	material constant
6	S_c_	0.8	material constant

**Table 2:** Orthogonal experiment table.

Levels	Alterable Hyperparameters		
	Initial Learning Rate	Batch Size	Numbers of Neurons in Hidden Layers
Case1	0.0001	8	120/100/80
Case2	0.001	16	100/80/60
Case3	0.01	32	80/60/40
Case4	0.1	64	60/40/20

**Table 3:** Results of orthogonal devised list.

Experimental Serial Number	Alterable Hyperparameters			MSE	Computing Time(s)
	Initial Learning Rate	Batch Size	Numbers of Neurons in Hidden Layers		
1	0.0001	8	120/100/80	6.404	486.50
2	0.0001	16	100/80/60	9.975	246.07
3	0.0001	32	80/60/40	11.514	115.57
4	0.0001	64	60/40/20	13.563	75.64
5	0.001	8	100/80/60	2.279	434.68
6	0.001	16	120/100/80	6.227	274.20
7	0.001	32	60/40/20	6.829	110.30
8	0.001	64	80/60/40	6.406	286.22
9	0.01	8	80/60/40	2.798	496.59
10	0.01	16	60/40/20	3.151	237.23
11	0.01	32	120/100/80	2.254	263.03
12	0.01	64	100/80/60	1.955	124.73
13	0.1	8	60/40/20	32.259	458.46
14	0.1	16	80/60/40	52.799	217.19
15	0.1	32	100/80/60	44.525	149.15
16	0.1	64	120/100/80	45.622	139.82

**Table 4:** The evaluation indicators of constitutive models.

Constitutive Model	Evaluation Indicators	
	C_C_	AARE
PB model	0.9989	4.681%
GRU model	0.9996	2.065%
Improved HSC model [48]	0.989	4.58%
LSTM model [48]	0.998	2.16%

## Data Availability

The raw/processed data acquired in present investigation cannot be shared at this time because the data also forms part of an ongoing research.
